# Immune gene signature delineates a subclass of thyroid cancer with unfavorable clinical outcomes

**DOI:** 10.18632/aging.102963

**Published:** 2020-04-02

**Authors:** Jingtai Zhi, Jiaoyu Yi, Mengran Tian, Huijuan Wang, Ning Kang, Xiangqian Zheng, Ming Gao

**Affiliations:** 1 Department of Thyroid and Neck Tumor, Tianjin Medical University Cancer Institute and Hospital, National Clinical Research Center for Cancer, Key Laboratory of Cancer Prevention and Therapy, Tianjin’s Clinical Research Center for Cancer, Tianjin 300060, People’s Republic of China

**Keywords:** tumor microenvironment, bioinformatics, immune checkpoint, TCGA

## Abstract

Thyroid cancer (THCA) is a heterogeneous disease with multiple clinical outcomes Immune cells regulate its progression. Three immunomolecular subtypes (C1, C2, C3) were identified in gene expression data sets from TCGA and GEO databases. Among them, subtype C3 had highest frequency of BRAF mutations, lowest frequency of RAS mutations, highest mutation load and shorter progression-free survival. Functional enrichment analysis for the genes revealed that C1 was up-regulated in the ERK cascade pathway, C2 was up-regulated in cell migration and proliferation pathways, while C3 was enriched in body fluid, protein regulation and response to steroid hormones functions. Notably, the three molecular subtypes exhibit differences in immune microenvironments as shown by timer database and analysis of immune expression signatures. The abundance of B_cell, CD4_Tcell, Neutrophil, Macrophage and Dendritic cells in C2 subtype were lower than in C1 and C3 subtypes Leukocyte fraction, proliferation macrophage regulation, lymphocyte infiltration, IFN gamma response and TGF beta response scores were significantly higher in C3 compared with C1 and C2 subtypes. Unlike C3 subtype, it was observed that C1 and C2 subtypes were significantly negatively correlated with most immune checkpoint genes in two different cohorts. The characteristic genes were differentially expressed between cancer cells, adjacent tissues, and metastatic tissues in different cohorts. In summary, THCA can be subclassified into three molecular subtypes with distinct histological types, genetic and transcriptional phenotypes, all of which have potential clinical implications.

## INTRODUCTION

Thyroid cancer (THCA) is the most common endocrine cancer and its global incidence has risen rapidly [1, 2]. In the United States, the annual incidence rate of THCA is 6.6% [3], which is the highest among all cancers. Despite the low death rate associated with thyroid cancer, its recurrence or progression rates are high, increasing morbidity and mortality rates in patients with THCA [4]. THCA has multiple histological types and subtypes affecting different cells, with distinct characteristics and prognosis [5]. Endocrine thyroid cells comprise follicular thyroid cells and parafollicular C cells. The follicular thyroid cell type has been implicated in papillary thyroid carcinoma (PTC), follicular thyroid carcinoma (FTC), poorly differentiated thyroid Cancer (PDTC) and anaplastic thyroid cancer (ATC). Although most thyroid cancers are not malignant, existing treatments do not sufficiently improve prognosis of patients with locally advanced or distant metastatic thyroid cancer. This has necessitated the search for highly sensitive therapies such as immunotherapy [6, 7]. In addition, several immune-related parameters have been proposed for prognosis prediction in patients with THCA [8, 9], indicating that different immune status is important in THCA prognosis. Therefore, understanding the immunophenotypes of the THCA microenvironment will promote the effective application of immunotherapy in THCA.

In human, the thyroid gland is the largest endocrine organ and is a common target for autoimmune diseases. Studies have shown that chronic lymphocytic thyroiditis (a common autoimmune disease) may trigger or accelerate the development of PTC [10, 11]. In *vivo*/*vitro* experiments suggest that immunological checkpoint inhibitors can eliminate thyroid tumor cells. A study found that BRAFV600E expression is positively correlated with PD-L1/PD-1 in PTC tissues, suggesting that immunological checkpoint inhibitors may be effective in PTC patients with BRAFV600E mutations [12]. In addition, immune cells are widely distributed in the thyroid cancer microenvironment [13], forming different tumor microenvironments (TME) in different stages of tumor development. Further, some immune cells can promote or inhibit tumorigenesis [14]. Currently, the role of immune cells in THCA is not fully known.

The identification of a specific thyroid cancer type is critical to the prognosis and treatment options of this malignancy. Previously, two molecular subtypes of PTC, namely BRAF V600E and RAS, were proposed based on transcriptome analysis of the cancer genome atlas (TCGA) database [15]. Recently, Seong-keun et al. [16] proposed a third molecular subtype (non-braf-non-ras) associated with follicle-patterned thyroid cancer. These reports show that the molecular phenotypes of thyroid cancer (TC) provide a better classification than the histological features. Many gene expression-based algorithms have been proposed for feature selection or molecular typing [17]. Among them is the CIBERSORT which uses linear support vector regression to infer the relative abundance of 22 immune cell subsets in tumors [18]. A similar algorithm for immunological backgrounds can facilitate immunological profiling of multiple cancer types [19]. In these approaches, a small number of "metagene signatures" are identified from gene expression profiles using negative matrix factorization (NMF) [20]. These studies indicate that methods for cancer gene expression profiling infers the abundance of matrix components [21] and tumor cell classification based on immune cell abundance [22] is feasible.

In this study, gene expression profiles of 781 THCA samples were obtained from the TCGA and Gene Expression Omnibus (GEO) databases. In addition, datasets of adjacent normal thyroid tissues and immune-related gene sets were obtained from ImmPort (https://immport.niaid.nih.gov). Thyroid immune gene expression profiles were analysed using NMF which identified three immune-related molecular subtypes with distinct characteristics. The immune characteristics, genomic features and clinical features of the three subtypes were systematically analyzed and a method for quantifying immune molecule subtypes, gSig score was then established. The gSig score showed high performance in predicting the prognosis of patients with THCA following treatment with immune checkpoint inhibitors.

## RESULTS

### Deconvolution of THCA immune related genes expression profiles into key molecular subtypes

**Figure 1:**
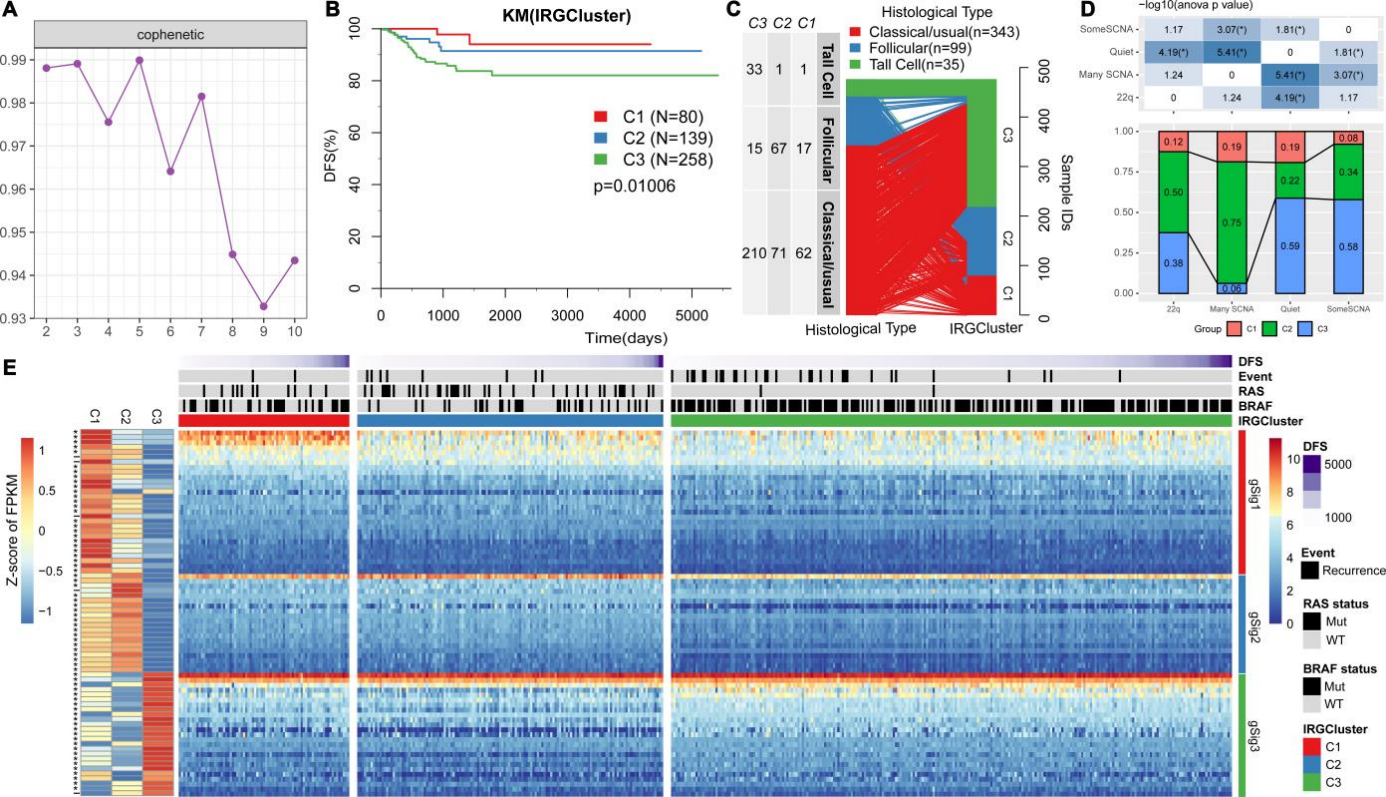
**IRGs-based THCA immunophenotyping.** (**A**) The interaction correlation coefficient (y-axis) is plotted against the number of subtypes (2-10; x-axis). Stability decreased between 3 and 4 subtypes, indicating that there are at least three molecular features in the expression profile of TCGA THCA IRGs. (**B**) KM curve showing the progression-free survival of each immunotype. (**C**) Comparison of pathological subtypes with IRGCluster. (**D**) Comparison of molecular subtypes with copy number variation and IRGCluster. The top panel is the heat map showing significant difference in the distribution of IRGCluster in the four subtypes with copy number variation. Bottom panel shows the distribution of IRGCluster in the four subtypes with copy number variation. (**E**) Heatmap showing THCA immunotypes and gene module gene expression, left panel is 74 genes on IRGCluster average expression level, * indicates FDR <0.05.

NMF clustering of 477 RNAseq-based gene expression profiles downloaded from the TCGA consortium was performed to identify key immune molecular subtypes underlying the heterogeneous THCA Related Genes (IRGs) expression profiles in reduced dimensions [15]. Specifically, we analyzed 705 high-expressed specific IRGs by univariate survival analysis. Consequently, we identified 74 genes which did not predict the progression-free survival of patients (logrank p<0.05). The NMF algorithm identified three optimal molecular subtypes in the IRGCluster; C1 (N=80), C2 (N=139), and C3 (N=258). A plot of cophenetic correlation coefficients, a measure of stability across the number of molecular subtypes examined, showed that the three molecular subtypes were present in the expression profiles (Figure 1A). A significant difference in progression-free survival among the three subtypes was observed (Figure 1B), with C3 subtype having the worst prognosis. In terms of distribution of THCA in the three pathological types (Figure 1C), Tall Cell variant was mainly distributed in C3 subtype (94.3%), while follicular was mainly distributed in C2 subtype (67.7%), suggesting different pathological THCA types have distinct immune characteristics. We then compared the IRGCluster with the previously proposed four molecular subtypes based on copy number variation (22q, Many SCNA, Quiet, SomeSCNA) [15]. Significant differences were observed in the IRGCluster distribution among the four subtypes (Figure 1D). The C3 subtype had the lowest proportion in many SCNA, the C1 subtype had the lowest proportion in SomeSCNA compared with the C2 subtype. Quiet had the lowest proportion in Quiet. These results indicate that the three molecular subtypes significantly overlap with existing molecular subtypes. Notably, some SCNA mainly contained C2 (34%) and C3 (58%) subtypes, which were enriched with BRAF mutations and were significantly associated with THCA clinical stage. In addition, C90 samples were enriched with > 90% BRAF mutations, and C2 samples had > 30% BRAF mutations. C3 subtype showed high invasion, high lymph node metastasis and advanced tumor stage compared withC1 and C2 (Supplementary Figure 1), suggesting that the IRGCluster-based molecular subtypes can be used for molecular stratification of patients based on their prognosis. The unsupervised clustering method was used to cluster the 74 gene expression profiles. Three types of gene sets, gSig1 (N=29), gSig2 (N=20) and gSig3 (N=25) were obtained. These three gene set types showed different expression patterns in the three molecular subtypes; gSig1 had the highest expression level in C1 and lowest expression level in C3, gSig2 was highly expressed in C1/C2 and weakly expressed in C3, gSig3 showed the highest expression in C3, but lower expression in C1/C2 (Figure 1E). Among these genes, 69 (93.2%) were differentially expressed among the three subtypes (FDR<0.05), and the BRAF mutations in C3 were significantly higher than in C1/C2 subtypes. However, RAS (NRAS/HRAS/KRAS) showed low expression levels in C3 compared with C1 and C2 subtypes (FDR<0.05). Studies have shown that BRAF mutations are highly associated with the occurrence and poor prognosis of the classical type of PTC, similar to our results.

### IRGCluster characteristic gene score and functional analysis

**Figure 2:**
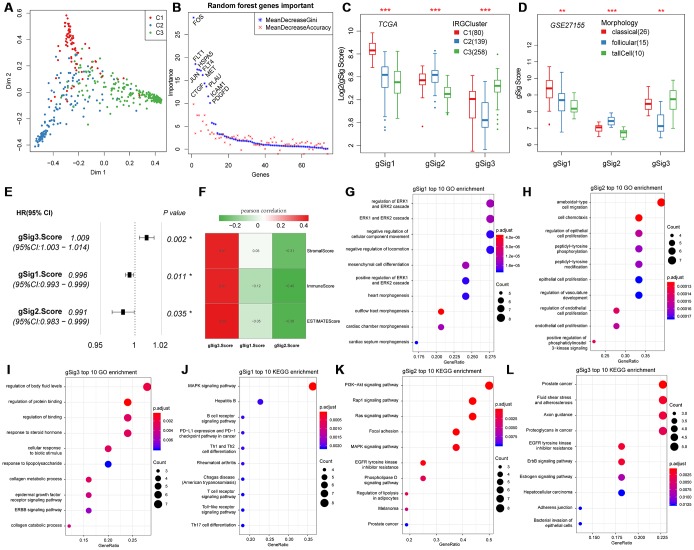
**Expression characteristics and functional annotation of the immune gene modules in each subtype.** (**A**) First and second principal component scores for each sample. (**B**) The importance ranking of 74 genes in random forest. (**C**) Distribution of gSigs score in IRGCluster. (**D**) Distribution of gSigs score in different pathological subtypes of GSE27155 data set. (**E**) The prognosis relationship between gSigs score and DFS. (**F**) Heat map showing gSig score and Pearson correlation results of immune infiltration. (**G**–**I**) GO enrichment analysis results of gSig1, gSig2 and gSig3. (**J**–**L**) KEGG pathway enrichment analysis results of gSig1, gSig2 and gSig3. Dot plot shows the top 10 GO and KEGG pathway enrichment results. The color in the graph indicates significance, and the dot size indicates the number of genes.

Principal component analysis was used to classify expression of the 74 genes into three subtypes (Figure 2A). The 74 genes were sequenced in importance through a random forest, we can see that there is a significant jump in the mean decrease gini index of top10 gene (Figure 2B), thus top10 genes were selected for subsequent analysis. In the top 10 important gene subsets, 3 gSig1genes, 4 gSig2 genes and 3 gSig3 genes were selected, and the gSig score for each group was presented as the average expression (Figure 2C). In terms of distribution among the subtypes, gSig1 was highly expressed in C1, gSig2 was highly expressed in C2, and gSig3 was highly expressed in C3 (Figure 2C). A set of external datasets GSE27155 were employed to verify this phenomenon (Figure 2D). Notably, we observed significant prognostic differences in DFS among the gSig scores in each sample (Figure 2E). Specifically, gSig3 was found to be a risk factor, while gSig1 and gSig2 were protective factors. The R software package ESTIMATE was used to calculate and compared the immune infiltration score of each sample. The gSig3 score was highly positively correlated with immune infiltration, while the gSig2 score was negatively correlated with immune infiltration (Figure 2F), suggesting that the gSig score can monitor the efficacy of immunotherapy. GO and KEGG enrichment analyses showed that gSig1, gSig2 and gSig3, were enriched in various biological pathways (Figure 2G-I). gSig1 was associated with ERK cascade (Figure 2G, Figure 2J), gSig2 was involved in cell migration and proliferation (Figure 2H, Figure 2K), gSig3 was associated with body fluid and protein regulation (Figure 2I, Figure 2L). The response to steroid hormone was significantly enriched in the gSig3 GO enrichment results (Figure 2I), which is significantly different from the response to steroid hormone seen in the extrathyroidal extension type of THCA in previous studies [23]. These results suggested that these gene sets are involved in different biological pathways which are associated with different clinical outcomes in patients with THCA.

### Association of immune gene sets of IRGCluster with metastasis, primary tumor, and adjacent tissues

**Figure 3:**
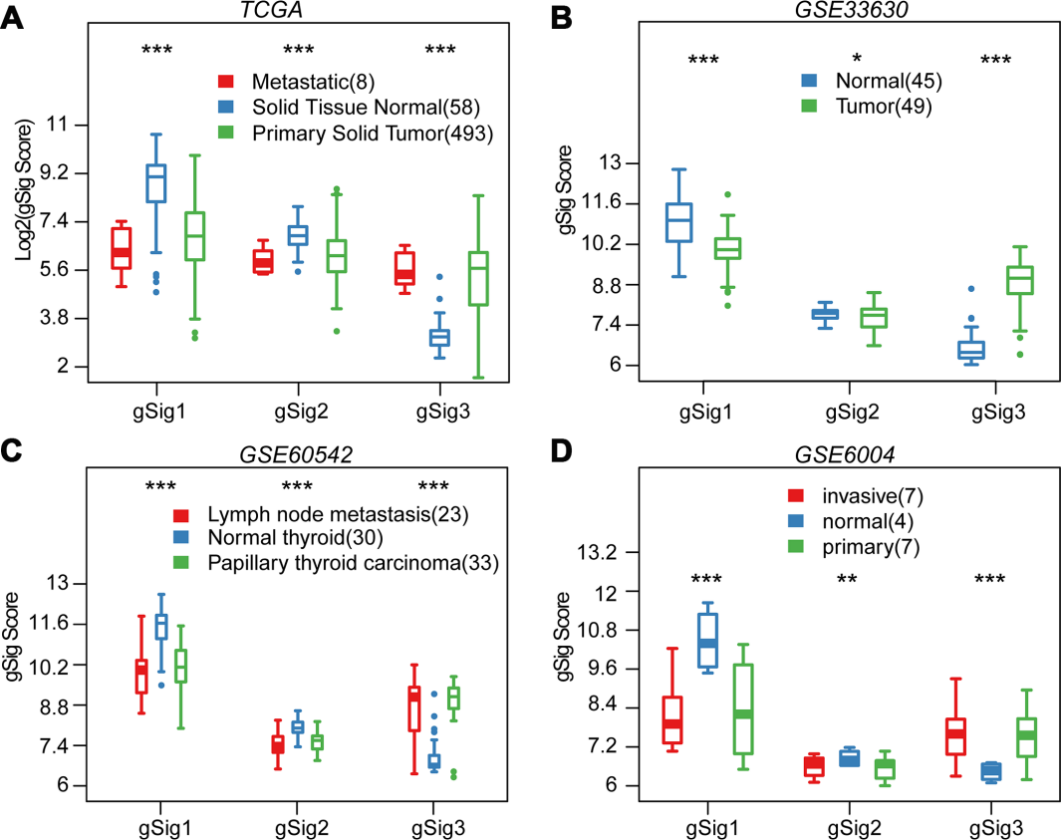
**Distribution of gSig score in different tissues.** (**A**) Distribution of gSig score in metastatic/normal/primary tissues in TCGA dataset. (**B**) Distribution of gSig score in normal/primary tissues in GSE33630 dataset. (**C**) Distribution of gSig score in metastatic/normal/PTC tissues in GSE60542 dataset. (**D**) Distribution of gSig score in invasive/normal/primary tissues in GSE6004 dataset.

We first evaluated the distribution of T, N, M, Stage, Age, and Lymph Node Counts in the three molecular subtypes (Table 1). Significant differences in T, N, Stage, and Lymph Node Count were observed among the three molecular subtypes (p<0.01). Advanced samples such as T, N, Stage and Lymph Node Count samples were highly distributed in the C3 subtype with the worst prognosis. This indicated that these three molecular subtypes are closely related to the clinical stage. The expression distribution of the three subtypes of characteristic gene scores in metastatic samples, primary samples and paracancerous samples were analyzed, and the three subtype characteristic gene scores showed extremely significant expression differences in different samples types (Figure 3A). The expression in the paracancerous samples in gSig1 and gSig2 was higher than that in the tumor samples, and the paracancerous samples in gSig3 were significantly lower than the tumor samples. A similar phenomenon was observed in the external datasets GSE33630, GSE60542, GSE6004 (Figure 3B–3D). These results indicated that the three subtypes of characteristic gene scores are markers of the THCA development and progression.

**Table 1:** Comparison of clinical features and IRGCluster.

	**C1**	**C2**	**C3**	***Chisq test***
pT				<0.001
T1	24	51	65	
T2	37	54	69	
T3	18	34	103	
T4	0	0	20	
Un	1	0	1	
pN				<0.001
N0	39	91	91	
N1	8	7	39	
N1a	11	12	61	
N1b	11	8	49	
Un	11	21	18	
pM				0.517
M0	47	65	158	
M1	0	2	4	
Un	33	72	96	
Stage				<0.001
I	51	88	133	
II	9	27	14	
III	16	19	69	
IV	4	4	41	
Un	0	1	1	
Age				0.159
0~50	52	79	133	
50~60	15	24	61	
60~70	5	24	35	
70~100	8	12	29	
Lymph Node Count				0.0024
0~5	26	54	83	
5~10	12	22	45	
10~20	7	3	39	
20~120	13	12	50	

### Immune microenvironment characteristics of Comparison of clinical features with IRGCluster

**Figure 4:**
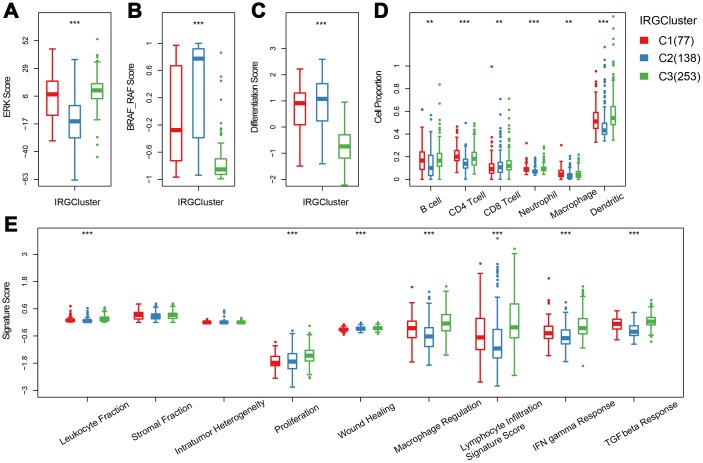
**The immune landscape of THCA IRGCluster.** (**A**–**C**) Distribution of ERK score, BRAF_RAF score and differentiation score in IRGCluster. (**D**) Six immune cell scores of IRGCluster. (**E**) Immune expression signature score of ERK score, BRAF_RAF score and differentiation score.

Previous studies have reported thyroid typing based on TCGA. Agrawal N [15] identified three molecular subtypes of thyroid papillary carcinoma; ERK score, BRAF_RAF score and differentiation score. In this study, the distribution of ERK score, BRAF_RAF score and differentiation score in the three immune subtypes was explored (Figure 4A–4C). The ERK score was significantly lower in C2 than that in C1/C3, and the BRAF_RAF and differentiation scores were significantly higher in C1/C2 than in C3. BRAF_RAF and the differentiation scores predicted poor prognosis, which is consistent with the better C1/C2 prognosis than C3. We further analyzed the differences in distribution of six immune cell components (B_cell, CD4_Tcell, CD8_Tcell, Neutrophil, Macrophage and Dendritic) in each subtype. Significant differences were observed in the distribution of the six immune cells among the three molecular subtypes. Notably, B_cell, CD4_Tcell, Neutrophil, Macrophage, and Dendritic were significantly lower in C2 than in C1 and C3, indicating that C2 was mainly enriched in follicular type samples. Although C1 and C3 were similar, CD4 T cell score was higher in C1 than C3, while CD8 T cell and dendritic cell scores were higher in C3 that in C1, suggesting distinct immune responses among the IRGCluster. A previous analysis showed that immune expression signatures [24] were significantly different between C2 and C1/C3 (Figure 4E). However, in C3 subtype with the worst prognosis, the leukocyte fraction, proliferation macrophage regulation, lymphocyte infiltration, IFN gamma response and TGF beta response scores were significantly higher than in C1/C2 subtypes. These results indicated that the three molecular subtypes have different immune microenvironments, which may underlie the differences in prognosis among them.

### Relationship between immune gene sets and expression of immune checkpoint genes in IRGCluster

**Figure 5:**
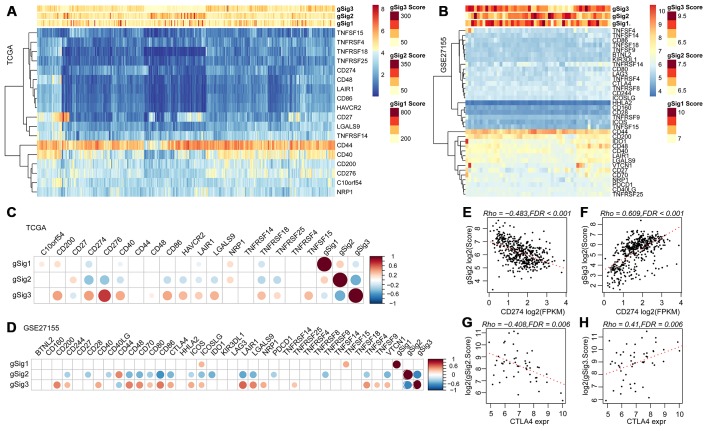
(**A**, **B**) Relationship between ICGs expression and gSig scores in the TCGA and GSE27155 data sets. (**C**, **D**) Correlation matrix of ICGs expression and gSig score in the TCGA and GSE27155 datasets. (**E**–**F**) Dot map showing CD274 gene expression, gSig2, and gSig3 in TCGA dataset. (**G**–**H**) Dot map showing GLA27155 gene expression, gSig2, and gSig3 in GSE27155 dataset. Correlation analysis used the spearman correlation coefficient.

We further analyzed 18 high-level immune checkpoint genes (ICGs) from the past literature to assess the relationship between the expression levels of these 18 ICGs and gSig1, gSig2 and gSig3 in the TCGA data set (Figure 5A). Overall, gSig1 and gSig2 were negatively correlated with the expression of 18 ICGs, while gSig3 was positively correlated with ICGs expression. The same phenomenon was observed in the external independent verification set GSE27155 (Figure 5B), gSig2 was significantly correlated with 11 (61.1%) ICGs, of which 9 (50%) were highly negatively correlated. gSig3 was significantly correlated with 13 (72.2%) ICGs, of which 12 (66.7%) were negatively correlated (Figure 5C), and similar results were also observed on the independent data set GSE27155 (Figure 5D). A recent study showed that CD274 (PD-L1) and CTLA4 are molecular markers of immunological checkpoint inhibitors. In the TCGA dataset, CD274 was highly expressed, the lower the gSig2, while the gSig3 is opposite (Figure 5E, 5F) (where CTLA4 is not detected). A similar phenomenon was observed in the GSE27155 set (Figure 5G, 5H) (wherein CD274 was not detected). The expression of CD274 and CTLA4 can be used to evaluate the benefit of immune checkpoint inhibitor therapy. Here, the samples were divided into benefits and non-benefit groups based on CD274 and CTLA4 expression. The gSig2 score and gSig3 score were used to predict the average AUC > 0.77 in CD274 expression sample from the TCGA data set. For CTLA4 expression in the GSE27155 data set, the average AUC > 0.73 (Supplementary Figure 2A), suggesting that gSig3 score may be a powerful prognostic biomarker and predictor of immune checkpoint inhibitor response. Similarly, the correlation between gSig score and the expression of known markers BRAF was analyzed. Notably, gSig1 and gSig2 showed a significant positive correlation with BRAF while gSig3 was significantly negatively correlated with BRAF (Supplementary Figure 2B). These results indicated that IRGCluster molecular subtypes can be used as immunotherapeutic molecular markers.

### Analysis of genomic heterogeneity in IRGCluster

**Figure 6:**
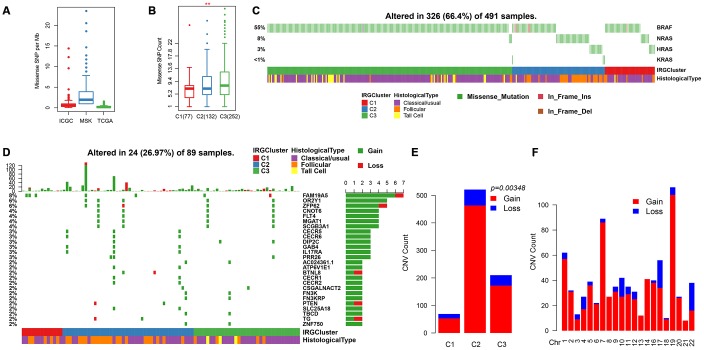
**Genomic mutations in IRGCluster.** (**A**) Quantity distribution of missense mutation (count per Mb) in ICGC, MSK and TCGA data sets. (**B**) Quantity distribution of mutations in IRGCluster sample. (**C**) BRAF and RAS gene mutations distribution. (**D**) CNV distribution in the top 27 gene. (**E**) CNV distribution in the IRGCluster sample. (**F**) Genomic distribution of CNV. Considering the instability of the INDEL detection, only the missense type mutation site is selected here.

Previous studies have shown that the overall mutation load of patients with THCA is low. The mutation load of ICGC (PTC-based), MSK (ATC/PDTC) and TCGA (PTC/FTC-based) data sets were compared. Result showed that the TMB of different pathological types with THCA patients were mainly concentrated below 5 (Figure 6A). In addition, we found that the mutation load in C3 was significantly higher than in C1/C2 (Figure 6B), suggesting that the mutation load is associated with poor prognosis. The distribution of BRAF and RAS (NRAS/HRAS/KRAS) genes with the highest mutation frequency on THCA in IRGCluster was analyzed and showed that the BRAF gene associated with poor prognosis had significantly higher mutations in C3 compared with C1/C2, whereas RAS mutations were higher in C1/C2 (Figure 6C). The NetworkAnalyst [25] tool was used to identify the pathways associated with BRAF. The five most significant pathways were Thyroid cancer (p = 0.00478), Bladder cancer (p = 0.0053), Endometrial cancer (p = 0.0075), Long-term depression (0.007755), Acute myeloid leukemia (0.00853), all of which were related to tumor invasion and metastasis, especially Thyroid cancer abnormalities. Further, the pathways involved in the RAS gene were analyzed, and the five most significant pathways were identified including Thyroid cancer (p = 1.01e-7), Bladder cancer (p = 1.38e-7), Endometrial cancer (p = 4e-7), VEGF signaling pathway (p = 4.21e-7), Long-term depression (4.43e-7), and the overlap of four pathways with BRAF, which indicates that these genes play a key role in the cancer development. However, the difference is that the RAS gene affects the VEGF signaling pathway. VEGF signaling pathway is related to neovascularization in tumors and may inhibit maturation and differentiation of dendritic cells and promote tumor progression. A total of 700 genes for Gain/Loss (688/112) were identified by CNV analysis, and these CNVs appeared on 89 samples (Figure 6D). Similarly, the frequency of CNV occurrence on THCA was low (median CNV count: 1), with only 27 genes having CNV in 2 or more samples (Figure 6D). GO was performed on these 27 genes, and the genes were enriched in the cellular and metabolic processes (Supplementary Table 1), while the KEGG pathway showed no significant enrichment results. Analysis of the relationship between IRGCluster and CNV showed that CNV was highly associated with C2 (Figure 6E), while the genomic distribution was significantly high on chr19 and chr7 (Figure 6F). These results indicated that different IRGCluster molecular subtypes have different genomic abnormalities.

## DISCUSSION

Tumor immunotherapy has emerged as a successful treatment for advanced tumors [26, 27]. A comprehensive understanding of THCA requires not only attention to tumor cells, but also understanding of the tumor microenvironment (TME) [28, 29]. The TME consists of a diverse group of cells that interact with cancer cells and participate in all stages of tumorigenesis. Immune cell infiltration in the tumor microenvironment has received much attention from researchers and has become a promising therapeutic target. Further research is required to decipher the immune signatures associated with the development and progression of THCA. This is especially important for the development of combination therapies. In this study, three molecular subtypes with clinical relevance were identified based on immune genes. We also predicted the prognosis of patients with THCA after initial treatment. The three molecular subtypes displayed different genomic characteristics and immune phenotypes. Moreover, the molecular subtypes were closely related to the expression of immune checkpoint genes and may have the potential of immunological checkpoints to block the therapeutic benefit.

Therapeutic antibodies that block the PD-1 / PD-L1 pathway can induce robust and long-lasting responses in patients with various cancers, including THCA [30–32]. However, these reactions occur only in a small number of patients, and some studies have found PD-1 expression, PD-L1 expression, MSI status and mutation load are not effective biomarkers for predicting the benefit of immune checkpoint blockade [33]. Therefore, it is important to develop biomarkers for predicting the benefits of checkpoint immunotherapy. In this study, we identified three molecular subtypes based on immune genes. These three molecular subtypes have different immune microenvironments, and the genes specifically expressed by the three subtypes are significantly correlated with the expression of most immune checkpoint genes. Genomic heterogeneity analysis revealed that the mutation load of the worst prognosis subtype C3 was significantly higher than the other two subtypes. These results suggest that the three molecular subtypes may have different response patterns to immunotherapy, and the characteristic genes of these three subtypes can be used as molecular markers to monitor responses to immunotherapy.

Comprehensive analysis showed that the three molecular subtypes reported by IRGCluster have different molecular characteristics and are prognostic biomarkers of THCA. C3 subtype is associated with poor prognosis. T, N, Stage and Lymph Node Count are key prognostic factors in THCA [34]. As expected, there was a significant association between IRGCluster and the patient's clinical features. Advanced T, N, Lymph Node Count, and Stage samples tended to be distributed in the worst prognostic C3 subtype. The expression distribution of the three subtypes of characteristic gene scores in metastatic, primary tumor and paracancerous samples. The expression level of gSig1 and gSig2 in the paracancerous samples was higher than that in the tumor samples, while the expression of gSig3 in paracancerous samples was significantly lower than that in the tumor sample. These findings were verified in an external independent data set. These three gene scores are important markers for the occurrence and development of thyroid cancer. We compared these three molecular subtypes with the PTC molecular subtypes from TCGA (ERK score, BRAF_RAF score and differentiation score). We found that the ERK score was significantly lower in C2 than that in C1/C3. The BRAF_RAF score and differentiation score were significantly higher in C1/C2 than in C3. Moreover, BRAF_RAF and differentiation scores correlated with poor prognosis.

Although we used bioinformatics techniques to identify potential immune gene markers involved in the development of THCA in large samples, further validation should be performed in a prospective THCA cohort receiving immunotherapy to fully define the cutoff values. Secondly, given the heterogeneity of different tumor regions, more clinical factors should be included in the prediction model for higher accuracy. Thus, the bioinformatic results obtained here requires experimental verification.

In summary, we systematically evaluated the expression profiles of 1811 immune genes in 781 THCA samples. We identified three immunologically relevant molecular subtypes with different characteristics, and established quantitative immunological molecular subtype specificities. The gSig scores presented in this study are powerful biomarkers for predicting responses to immune checkpoint inhibitor therapy.

## MATERIALS AND METHODS

### Data collection and processing

THCA RNA-seq expression profile data was downloaded from TCGA database (https://portal.gdc.cancer.gov/) using GDC API (https://gdc.cancer.gov/developers/gdc-application-programming-interface-api). Data comprising 572 samples and the prognostic information of these samples were also downloaded on March 14, 2019. The specimen used were surgically removed before systemic treatment. Tumor and normal thyroid samples were obtained from patients with the approval from local institutional review boards. RNA-seq was performed using the Illumina TruSeq library construction protocol (non-stranded, polyA+ selection). Sequencing data was processed using GDC standard pipeline (https://docs.gdc.cancer.gov/Data/Bioinformatics_Pipelines/Expression_mRNA_Pipeline/) to obtain the FPKM expression profile data. Tumor samples without clinical data and those with a follow-up period of less than 30 days were excluded. Finally, 477 primary tumor samples, 8 metastatic samples, and 58 paracancerous samples were included in this study. Four sets of chip datasets GSE27155 [35], GSE33630 [36], GSE60542 [37], and GSE6004 [38] were downloaded from the Gene Expression Omnibus (GEO) database. The GSE27155 dataset was from the Affymetrix Human Genome U133A Array, the GSE33630, GSE60542, and GSE6004 dataset were from Affymetrix Human Genome U133 Plus 2.0 Arrays containing 99, 95, 92, and 18 samples, respectively. After initial surgical treatment, tumor progression was defined as the neonatal, metastatic and recurrence. For the probe data, the probe was mapped to the GeneSymbol using the R package hgu133plus2.db, and median was obtained when the multiple probes correspond to the expression of one gene. Probes matching multiple genes were removed. The sample statistics are shown in Table 2.

**Table 2:** Sample information for each data set.

**TCGA**	**Samples**	**classical**	**follicular**	**tall cell**	**other**
Metastatic	8				
Primary Solid Tumor	493				
DFS>=30 days	477	343	99	35	
Solid Tissue Normal	58				
GSE27155					
tumor	51	26	15	10	
normal	44				
GSE33630					
tumor	49	49			
normal	45				
GSE60542					
Lymph node metastasis	23	16	3		4
Normal lymph node	4	3	0		1
Papillary thyroid carcinoma	33	20	6		7
Normal thyroid	30	21	6		3
Pleural metastasis	1	0	0		1
Recurrence	1	1	0		0
GSE6004					
primary	7				
invasive	7				
normal	4				

The immune-related gene was derived from the ImmPort database (https://immport.niaid.nih.gov), and 1811 immune related genes (IRGs) were obtained after excluding the duplicates. The RNA-seq data was defined in 50% of the samples, and the FPKM < 1 gene was identified as a universally low expression gene and was excluded in this study.

### Identification of immune molecular subtypes (IRGCluster)

Nonnegative Matrix Factor (NMF) is an unsupervised clustering method that is widely used in the discovery of genomics-based tumor molecular subtypes [39, 40], and is a matrix decomposition method under the constraint that all elements in the matrix are non-negative. It has higher efficiency and less storage space when processing large-scale data, and it can minimize the reconstruction error of the original matrix while maintaining the statistical information of the original data. To examine the expression and phenotype of prognosis-related immune genes relationships, univariate survival analysis was performed to identify immune genes significantly associated with prognosis in THCA patients. Based on prognostic-related expression profiles of immune genes, the NMF method was used to re-cluster the samples and analyze the clinical features of the re-clustered samples The NMF method selects the standard "brunet" for 50 iterations, and the number of clusters k is set to 2-10, and the average profile width of the common member matrix is calculated using the R package NMF [41] The minimum member of each subclass was set to 10, and cophentic, dispersion, and rss indicators of K=2-10 are evaluated. The optimal number of clusters was selected based on these three indicators.

### Characteristic genes evaluable of IRGCluster

To select the characteristic genes of the three immune molecular subtypes, the unsupervised clustering method was used to classify the genes with significant prognosis, and the expression patterns of each classification were further analyzed. Random forest algorithm was used for dimensionality analysis to assess the importance of each immune gene. First, the number of random variables (mtry parameters) for each segmentation was set as 1-74, ntree as 500 and the mtry value with the lowest error rate was selected as the optimal mtry value for the random forest algorithm, and ntree = 80 was selected according to the error rate of the random forest. Finally, each IRG was sorted based on importance and the top10 gene were selected as the subtype characteristic gene. The average expression level of the subtype-specific gene in the subtype-specific expression gene set was used as the subtype characteristic gene score (gSig score).

### The relationship of IRGCluster and clinical feature

To observe the relationship between IRGCluster and clinical phenotypes, the distribution of TNM, Stage, Age, and Lymph Node count information in the TCGA dataset samples was compared in the IRGCluster.

### The relationship of IRGCluster and immune microenvironment

To explore the relationship between IRGCluster and the immune microenvironment, the online TIMER tool [42] (tumor immune estimation resource) was used to calculate the six immune cell scores of THCA samples from TCGA using default parameters, and the differences of the six immune cell scores in different IRGCluster were analyzed. At the same time, the distribution of three molecular subtypes of TCGA Papillary Thyroid Carcinoma [24] in IRGCluster were compared and further the relationship between IRGCluster characteristic gene score and immune checkpoint genes was analyzed. A total of 47 immune checkpoint genes [43] were retrieved from the literature. The gene expression levels were screened, and the Spearman correlation of each immune checkpoint gene and the IRGCluster characteristic gene score was calculated.

### The relationship of IRGCluster and tumor genomic variation

The CNV data of THCA was downloaded from TCGA. First, the CNV intervals were merged using the following criteria:

1. 50% regional overlap in the two intervals is considered the same interval.
2. The number of coverage probes <5 intervals were removed.
3. The CNV interval was mapped to the corresponding gene using the GRh38 version of gencode.v22.
4. Multiple CNV regions in one gene region were combined into one, and average value as the combined CNV values.

SSNV mutation data were comes from three data sets: TCGA (https://www.cancer.gov/about-nci/organization/ccg/research/structural-genomics/tcga), ICGC (https://icgc.org/) and MSK-IMPACT (https://www.mskcc.org/msk-impact), in which TCGA and ICGC were sequenced for the whole exome, and MSK-IMPACT was for the 341 gene panel data. The number of missense mutations in each sample was calculated for Tumor Mutation Load Analysis. For the whole external sequencing, 38.4 Mb was taken as the genomic interval, and for the 341 gene panel, 1.023 Mb was taken as the genomic interval

### Functional enrichment analyses

Gene Ontology (GO) and Kyoto Encyclopedia of Genes and Genomes (KEGG) pathway enrichment analyses were performed using the R package clusterprofiler [44] for genes to identify over-represented GO terms in three categories (biological processes, molecular function and cellular component), and KEGG pathways. For these analyses, a FDR <.05 represented statistical significance.

### Statistical analysis

Univariate survival analysis was performed using the cox risk regression model, and log rank p < 0.05 was used as a cut off threshold to screen for immune-related genes (IRGs) that were significantly associated with prognosis. The Kaplan-Meier method was used to generate survival curves for the subgroups in each data set, and the log-rank test was used to determine the statistical significance of the differences, significance was defined as P < 0.05. The chisq test was used for overlapping samples between histological type and IRGCluster. The preference for the distribution of the case and clinical feature grouping samples on the IRGCluster was tested for significance. The two-group significance test for continuous variables used the Wilcox rank test, and the significance test for more than two subgroups used the Kruskal-Wallis rank test, the Benjamini-Hochberg method to convert the P values to FDR. The R 3.5.1 software was used for these analyses. Unless otherwise stated, *** indicates p < 1e-5, ** indicates p < 0.01, and * indicates p < 0.05.

## Supplementary Material

Supplementary Figures

Supplementary Table 1
