# Supplementary material for: Immune gene signature delineates a subclass of thyroid cancer with unfavorable clinical outcomes
Source: Aging (Albany NY). 2020 Apr 2;12(7):5733–50. doi: 10.18632/aging.102963 (PMC7185138; doi:10.18632/aging.102963)
Supplement: Supplementary Figures [file aging-12-102963-s002..pdf]

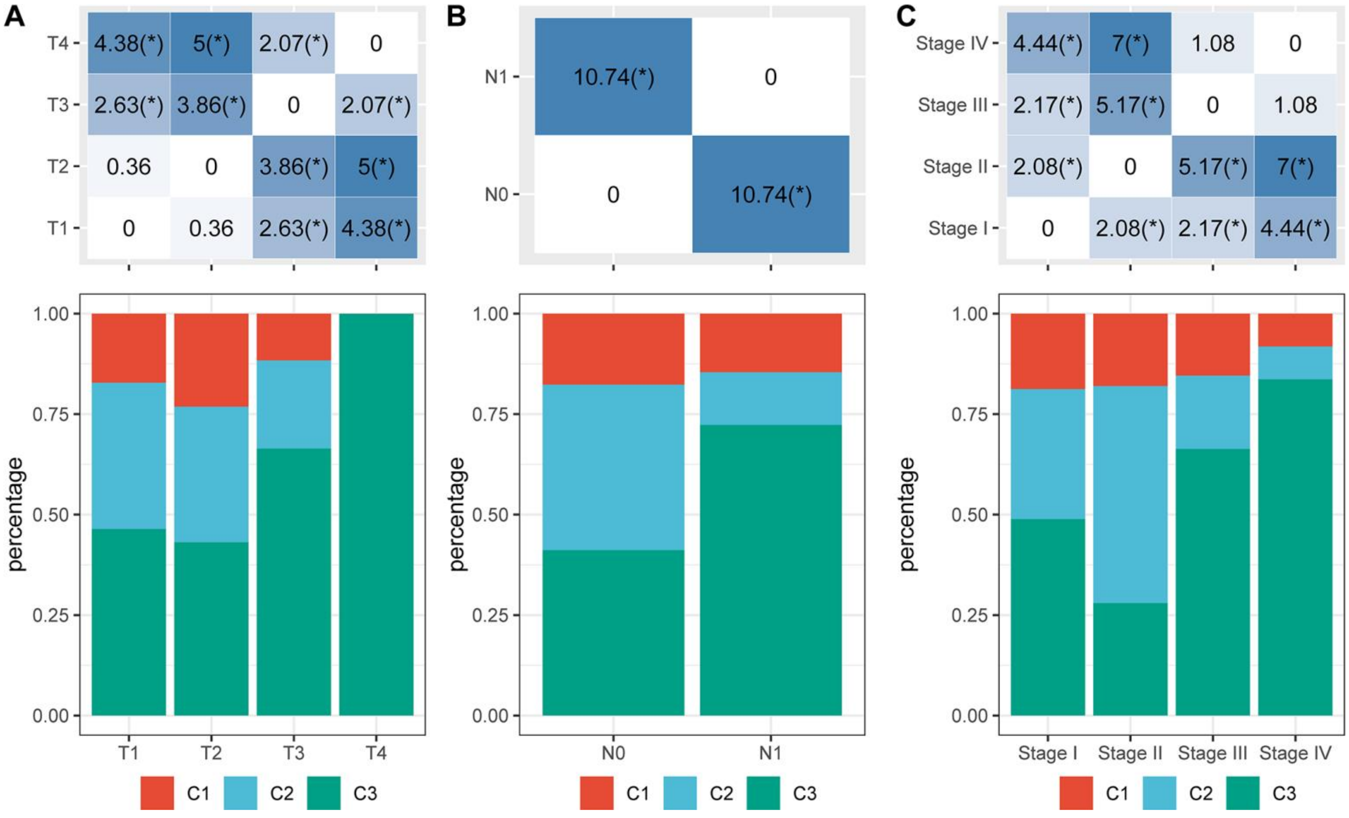

Supplementary Figure 1. IRGCluster was compared with clinical stage. (**A**–**C**) The top panel was the heat map of the significant difference in the distribution of IRGCluster in clinical stage T, N and stage. The significance p value was obtained by anova test, the median value was -log10 (p value), bottom panel was the distribution proportion of IRGCluster in clinical stage.

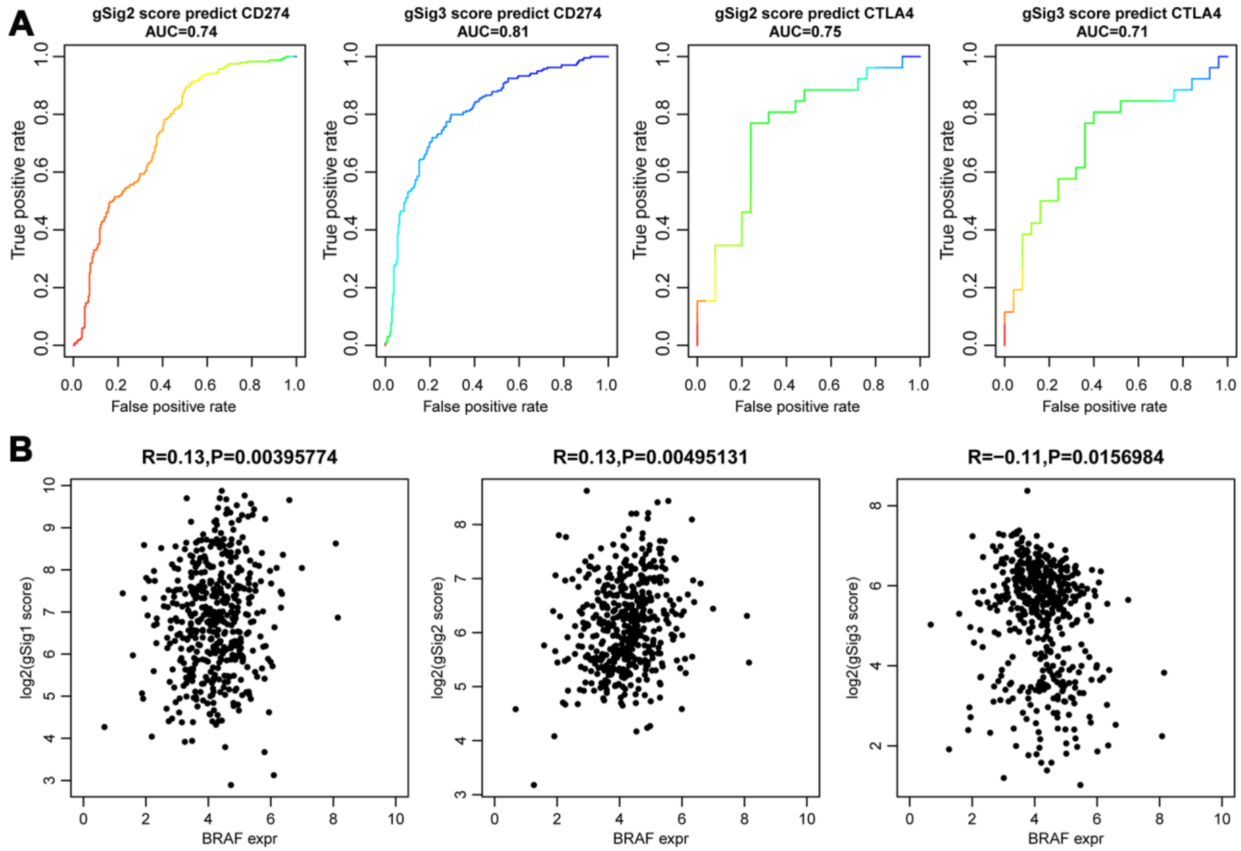

Supplementary Figure 2. (**A**) gSig2 score and gSig3 score predicted the AUC curve of high and low expression of CD274 in TCGA data set and the AUC curve of high and low expression of CTLA4 in GSE27155 data set. (**B**) correlation between three gSig scores and BRAF expression in TCGA data set.
