# Supplementary material for: Immune gene signature delineates a subclass of thyroid cancer with unfavorable clinical outcomes
Source: Aging (Albany NY). 2020 Apr 2;12(7):5733–50. doi: 10.18632/aging.102963 (PMC7185138; doi:10.18632/aging.102963)
Supplement: Supplementary Table 1 [file aging-12-102963-s001..pdf]

SUPPLEMENTARY TABLE

Supplementary Table 1. GO is performed on these 27 genes, and found that they were mainly concentrated in the cellular process and the metabolic process.

ID	Description	Count	GeneRatio	geneID
GO:0000003	reproduction	1	27-Jan	PTEN
GO:0008152	metabolic process	13	13/27	ZFP62/CNOT6/FLT4/MGAT1/ATP6V1E1/CECR2/CSGALNACT2/FN3K/FN3KRP/PTEN/SLC25A18/TG/ZNF750
GO:0001906	cell killing	0	0/27	
GO:0002376	immune system process	2	27-Feb	IL17RA/BTNL8
GO:0006791	sulfur utilization	0	0/27	
GO:0006794	phosphorus utilization	0	0/27	
GO:0040007	growth	2	27-Feb	SCGB3A1/PTEN
GO:0007610	behavior	1	27-Jan	PTEN
GO:0008283	cell proliferation	4	27-Apr	CNOT6/FLT4/SCGB3A1/PTEN
GO:0009758	carbohydrate utilization	0	0/27	
GO:0009987	cellular process	18	18/27	OR2Y1/ZFP62/CNOT6/FLT4/MGAT1/SCGB3A1/IL17RA/ATP6V1E1/BTNL8/CECR2/CSGALNACT2/FN3K/FN3KRP/PTEN/SLC25A18/TBCD/TG/ZNF750
GO:0015976	carbon utilization	0	0/27	
GO:0019740	nitrogen utilization	0	0/27	
GO:0022414	reproductive process	1	27-Jan	PTEN
GO:0022610	biological adhesion	2	27-Feb	PTEN/TBCD
GO:0023052	signaling	9	27-Sep	OR2Y1/CNOT6/FLT4/SCGB3A1/IL17RA/ATP6V1E1/BTNL8/PTEN/TG
GO:0032501	multicellular organismal process	8	27-Aug	OR2Y1/FLT4/MGAT1/IL17RA/CECR2/PTEN/TBCD/TG
GO:0032502	developmental process	9	27-Sep	FLT4/MGAT1/SCGB3A1/CECR2/FN3K/PTEN/TBCD/TG/ZNF750
GO:0040011	locomotion	3	27-Mar	FLT4/IL17RA/PTEN
GO:0043473	pigmentation	0	0/27	
GO:0044848	biological phase	0	0/27	
GO:0048511	rhythmic process	1	27-Jan	PTEN
GO:0048518	positive regulation of biological process	7	27-Jul	CNOT6/FLT4/SCGB3A1/IL17RA/BTNL8/PTEN/ZNF750
GO:0048519	negative regulation of biological process	5	27-May	CNOT6/FLT4/SCGB3A1/PTEN/TBCD
GO:0050789	regulation of biological process	12	27-Dec	OR2Y1/ZFP62/CNOT6/FLT4/SCGB3A1/IL17RA/ATP6V1E1/BTNL8/PTEN/TBCD/TG/ZNF750
GO:0050896	response to stimulus	10	27-Oct	OR2Y1/CNOT6/FLT4/SCGB3A1/IL17RA/ATP6V1E1/BTNL8/PTEN/SLC25A18/TG
GO:0051179	localization	7	27-Jul	FLT4/IL17RA/ATP6V1E1/CECR2/PTEN/SLC25A18/TG
GO:0051704	multi-organism process	2	27-Feb	IL17RA/PTEN
GO:0065007	biological regulation	12	27-Dec	OR2Y1/ZFP62/CNOT6/FLT4/SCGB3A1/IL17RA/ATP6V1E1/BTNL8/PTEN/TBCD/TG/ZNF750
GO:0071840	cellular component organization or biogenesis	5	27-May	CNOT6/SCGB3A1/CECR2/PTEN/TBCD
GO:0098743	cell aggregation	0	0/27	
GO:0098754	detoxification	0	0/27	
